# Population and size‐specific distribution of Atlantic salmon *Salmo salar* in the Baltic Sea over five decades

**DOI:** 10.1111/jfb.14213

**Published:** 2019-12-17

**Authors:** Philip Jacobson, Anna Gårdmark, Magnus Huss

**Affiliations:** ^1^ Department of Aquatic Resources Swedish University of Agricultural Sciences Öregrund Sweden

**Keywords:** anadromy, Atlantic salmon, Baltic Sea, body size, population‐specific management, spatial distribution

## Abstract

Population‐specific assessment and management of anadromous fish at sea requires detailed information about the distribution at sea over ontogeny for each population. However, despite a long history of mixed‐stock sea fisheries on Atlantic salmon, *Salmo salar*, migration studies showing that some salmon populations feed in different regions of the Baltic Sea and variation in dynamics occurs among populations feeding in the Baltic Sea, such information is often lacking. Also, current assessment of Baltic salmon assumes equal distribution at sea and therefore equal responses to changes in off‐shore sea fisheries. Here, we test for differences in distribution at sea among and within ten Atlantic salmon *Salmo salar* populations originating from ten river‐specific hatcheries along the Swedish Baltic Sea coast, using individual data from >125,000 tagged salmon, recaptured over five decades. We show strong population and size‐specific differences in distribution at sea, varying between year classes and between individuals within year classes. This suggests that Atlantic salmon in the Baltic Sea experience great variation in environmental conditions and exploitation rates over ontogeny depending on origin and that current assessment assumptions about equal exploitation rates in the offshore fisheries and a shared environment at sea are not valid. Thus, our results provide additional arguments and necessary information for implementing population‐specific management of salmon, also when targeting life stages at sea.

## 1 INTRODUCTION

Knowledge about spatial distribution patterns of fish populations is key to ensure that conservation and fisheries management actions effectively target the population in question. However, distribution is often highly heterogeneous across temporal and spatial scales, which can pose a great challenge and limit the use of such information in both assessment and management. The extent of variation in distribution differs both between species (*e.g*., sedentary *v*. migratory species) and among populations within species (Dunn & Pawson, 2002, Laikre *et al*., 2005, Ruzzante *et al*., 2006). Distribution patterns of fish can also be highly variable among individuals within populations; *e.g*., due to partial migration (Chapman *et al*., 2012, Jonsson & Jonsson, 1993) and ontogenetic habitat shifts leading to variation in habitat use among life stages (Barbeaux & Hollowed, 2018, Dahlgren & Eggleston, 2000, Werner & Gilliam, 1984). Within‐population variation in migration and distribution patterns is governed by a range of abiotic factors (*e.g*., temperature; Barbeaux & Hollowed, 2018, Morita *et al*., 2014, Otero *et al*., 2014), biotic factors (*e.g*., prey availability and predator avoidance; Brönmark *et al*., 2008, Barnett & Semmens, 2012) and genetics (Johnston *et al*., 2014, Barson *et al*., 2015). Within population variation in migration have implications for population dynamics (*e.g*., alternative stable states in stage‐specific abundance; Schreiber & Rudolf, 2008), trophic interactions (*e.g*., affecting the predation pressure of planktivorous fish on zooplankton, affecting plankton spring dynamics; Brodersen *et al*., 2011) and thus, ecosystem dynamics (*e.g*., affecting interaction strengths between and within trophic levels; Brodersen *et al*., 2008, Miller & Rudolf, 2011). Therefore, accounting for such variation is important to understand how changes in the experienced environment, including variation in exploitation rates and prey densities, affect the dynamics of heterogeneously distributed populations. Still, we often lack knowledge on distribution differences between and within populations, especially in large and open aquatic systems.

Knowledge on spatial distribution patterns is particularly important for anadromous species, where population dynamics is a consequence of the performance of individuals in both rivers and oceans (Chaput, 2012, Jensen *et al*., 2018, Moore *et al*., 2014). Management of anadromous fish may require actions targeting individuals in both habitats (Allen & Singh, 2016). For example, actions to increase survival at sea (Chaput, 2012) and preserve diversity among individuals across life stages (demographic structure) is important to ensure population stability (Moore *et al*., 2014, Schindler *et al*., 2010) and to stabilise fisheries yield (Schindler *et al*., 2010). One of the ecologically and economically most important anadromous fish species in the North Atlantic Ocean and in the Baltic Sea is the Atlantic salmon *Salmo salar* L. 1758 (Hindar *et al*., 2011, Kulmala *et al*., 2013). Variation in individual distribution during the feeding phase of Atlantic salmon at sea has been observed in the eastern and western North Atlantic Ocean, based on information from archival tags from repeat spawners (Lacroix, 2013b, Strøm *et al*., 2017, 2018), stable‐isotope signatures from scales (MacKenzie *et al*., 2012) and muscle tissue (Dempson *et al*., 2010). These studies suggest that the distribution of individuals at sea is more similar in some populations than in others and can also vary within populations depending on the sea age of individuals, which is partly governed by genetics (Barson *et al*., 2015, Johnston *et al*., 2014). However, these studies are based on few individuals from few populations and only take the distribution of repeat spawners or the distribution during the last growth season into account (but see Quinn *et al*., 2011, Shelton *et al*., 2019, Weitkamp & Neely, 2002 for studies on other anadromous salmonid species). In the Baltic Sea, differences in distribution have been observed among and within Finnish Atlantic salmon populations, based on tagged salmon recaptures and stable‐isotope analyses (Kallio‐Nyberg *et al*., 1999, Kallio‐Nyberg & Ikonen, 1992, Torniainen *et al*., 2013). These distribution differences have been linked to prey availability, differences in smolt size, origin (hatchery or wild) and genetics (Jutila *et al*., 2003, Kallio‐Nyberg *et al*., 1999, 2015, Salminen *et al*., 1994). Still, we have limited knowledge regarding how the distribution pattern of Atlantic salmon in the Baltic Sea (henceforth, Baltic salmon) varies over ontogeny, how the distribution varies among and within Swedish Baltic salmon populations and how temporally stable these distribution patterns are among and within populations.

Atlantic salmon at sea are exploited by mixed‐stock fisheries, as salmon from different populations aggregate and feed in similar geographic regions (ICES, 2017, Koljonen, 2006). Therefore, detailed knowledge on the population‐specific distribution of salmon at sea is important for estimating population‐specific harvesting rates (Crozier *et al*., 2004, Ruzzante *et al*., 2006, Whitlock *et al*., 2018). In the Baltic Sea, offshore mixed‐stock sea fisheries were long the dominant type of fisheries targeting Baltic salmon, but during the recent decades offshore fisheries have decreased (ICES, 2018, Karlsson & Karlström, 1994). Nowadays, Baltic salmon are mainly exploited by commercial and recreational coastal and river fisheries (ICES, 2018). These fisheries target returning adults on their spawning migration from their feedings grounds towards and within their natal rivers. Thus, river fisheries are population‐specific while the coastal fisheries still targets salmon from a mix of populations, but becomes increasingly population‐specific the closer the river mouth the fishing is conducted (Whitlock *et al*., 2018). Current assessment of Baltic salmon populations assumes that they have identical distribution at sea and thus, equal exploitation rates at sea in the offshore sea fisheries, while for the coastal fisheries, harvesting rates are assumed to be equal within assessment units (one assessment unit (AU; six in total) contains a group of Baltic salmon populations (ICES, 2015, 2018)). Whether these simplifying assumptions of equal harvesting rates at sea hold is not known, as we lack information on the distribution of Swedish Baltic salmon at sea and, for all Baltic salmon populations, how it varies over time and over ontogeny.

Here, we test how the distribution patterns of salmon at sea vary over ontogeny among and within 10 different salmon populations of hatchery origin feeding in the Baltic Sea, using data of >125,000 tagged, released and recaptured salmon covering the time period 1951–1999. We tested for variation in latitudinal distribution at different biological levels of organisation, including population, year class and individual‐level variation. We show strong population and size‐specific differences in both mean distribution of salmon at sea and variation in distribution between year classes and among individuals within year classes.

## 2 MATERIALS AND METHODS

The rearing practices and tagging (sedated salmon smolts tagged with external Carlin tags) procedures used in this study complied with Swedish animal welfare laws, guidelines and policies as approved by various authorities; *e.g*., the Swedish Board of Agriculture and water courts decisions legitimate for 1950–1999. The reported recaptures of tagged salmon caught at sea has been recaptured by various types of fisheries with the large majority by commercial fisheries. Thus, all recaptured and reported fish was killed. No additional experiments were carried out using the tagged fish.

### 2.1 Recapture data

To assess the distribution patterns of different salmon populations feeding in the Baltic Sea, we used recapture data from the Swedish tagging programme, initiated in 1951, in which a proportion of all reared salmon smolt are tagged with Carlin‐tags before release. These smolts are reared to compensate for the loss of natural salmon production in rivers with hydropower dams and to enhance wild populations with poor status (Karlsson & Karlström, 1994, Romakkaniemi *et al*., 2003). Carlin‐tags are external tags (attached below the dorsal fin), each having a unique serial number and instructions for reporting the catch (Supporting Information Figure S1). Length, origin, age, release location and date are recorded when the smolt is tagged. If a tagged individual is recaptured, the catcher is instructed to return the tag together with date, length, mass, type of fishing (recreational, commercial, brood stock or scientific), recapture location together with any additional comments. Until 1999, the Swedish Salmon Research Institute managed the database containing all releases and recaptures of tagged individuals, after which the hydropower companies have managed the database. After 1999, the recapture report rate, data quality and availability have decreased (ICES, 2013). For this study, we have managed to assemble recapture data from 1951–1999 (125,432 individuals with known origin, recapture location and size at recapture) with sufficient recaptures from 10 populations and recaptures from, 2004–2010 (418 individuals with known origin, recapture location and size at recapture from nine different populations, of which 192 recaptures originated from Luleälven (Supporting Information Figure S2)). To ensure that we assess the feeding distribution and not the distribution during the spawning migration towards their natal river, we excluded individuals that were caught in coastal gear types during the predominant spawning migration time (May–July; Siira *et al*., 2009, Whitlock *et al*., 2018), as well as all individuals caught in rivers all year around.

### 2.2 Recapture location

Each recaptured salmon with information about the recapture location has been given a corresponding recapture zone according to a specific map (Supporting Information Figure S3) when entered into the database. We converted these recapture zones to coordinates corresponding to the centre of each recapture zone using the World Geodetic System 1984 (WGS84; http://www.nga.mil/ProductsServices/GeodesyandGeophysics/Pages/WorldGeodeticSystem.aspx) decimal coordinate system.

### 2.3 Statistical analyses

We tested for size‐specific distribution differences among salmon at three levels of biological organisation; (a) differences in the mean latitudinal distribution among populations (population and size‐specific differences in latitudinal distribution); (b) differences in the latitudinal distribution variation of year‐classes; *i.e*., smolts released in the same year, between populations (differences in year‐class distribution variation among populations); (c) differences in the degree of individual latitudinal distribution variation within year classes among populations (individual variation in distribution).

#### 2.3.1 Population and size‐specific differences in latitudinal distribution

To test for size‐specific differences in the mean latitudinal distribution among populations, we calculated the annual mean recapture latitude for six size classes (10–30, 30–50, 50–70, 70–90, 90–110 and 110–130 cm) for each population and analysed for differences between populations and between size classes using two‐way ANOVA including population and size class as explanatory factors. We used Tukey's honest significant difference (HSD) *post hoc* test to determine which populations' distributions differed significantly.

#### 2.3.2 Differences in year‐class distribution variation between populations

To test for differences in year‐class distribution variation between populations, we used Levene's test for homogeneity of variance, comparing the annual variance of recapture latitudes within each size class among populations.

#### 2.3.3 Individual variation in distribution

To test for differences in individual variation in distribution, we compared the SD of the annual mean recapture latitude of each year class between populations using two‐way ANOVA including population and size class as explanatory factors. We used Tukey's HSD *post hoc* test to determine which population's distribution variation differed significantly.

**Figure 1:**
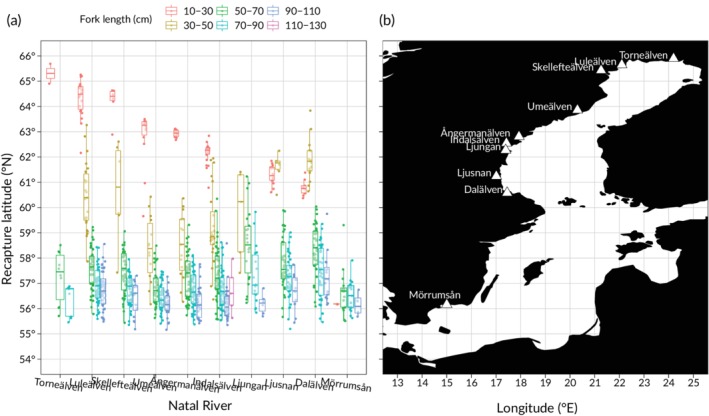
(a) Map showing the river outlet locations of the 10 Baltic *Salmo salar* tag recapture populations analysed (1951–1999) and (b) boxplots (−, median;, interquartile range; |, 95% range; •, outliers; each data point ≥10 recaptures) showing their population‐specific mean recapture latitude by fork‐length class, sorted north (left) to south (right) according to river location. Salmon size‐class (cm): (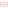) 10‐30, (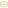) 30‐50, (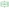) 50‐70, (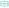) 70‐90, (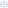) 90‐110, (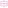) 110‐130

For all analyses, the smallest size‐class, 10–30 cm, was excluded as these recaptures are governed by the location of each populations' river and not by the distribution of individuals feeding at sea (Figure 1 and Supporting Information Figure S4). The largest size‐class, 110–130 cm, was excluded in all statistical analyses due to insufficient sample sizes to compare distribution among populations (Figure 1 and Supporting Information Figure S4). We also excluded distribution estimates based on <10 recaptured individuals within a size class of a specific year class or recapture year and recaptures from, 2004–2010 in all analyses due to the low number of recaptures (418 individuals with known origin, recapture location and size at recapture from nine different populations, of which 192 recaptures originated from Luleälven; Supporting Information Figure S2 and Table S1). Validity of assumptions on homogenous variance, normally distributed residuals and potential outliers were assessed visually using quantile–quantile (QQ), residual *v*. fitted and residual‐leverage plots. All statistical analyses were conducted using R 3.5.1 (R Core Team, 2018). We performed Levene's test for homogeneity of variance using the function **leveneTest**(), included in the R‐package Car, 3.0–2 (Fox *et al*., 2018).

## 3 RESULTS

### 3.1 Population and size‐specific differences in latitudinal distribution

Generally, all 10 Baltic salmon populations migrated to the southern Baltic Sea to feed after leaving their respective natal river (Figure 1). However, the latitudinal extent of this southward migration differed between populations (ANOVA, *F*_1,9_ = 33.642, *P* < 0.001) and size‐classes (ANOVA, *F*_1,3_ = 369.008, *P* < 0.001). The size‐dependency of this southward migration also differed between populations (interaction term, ANOVA, *F*_1,24_ = 5.075, *P* < 0.001; Figure 1 and Supporting Information Figure S4). In size class 30–50 cm, salmon from Umeälven and Ångermanälven were caught furthest south and salmon from Ljusnan and Dalälven mostly to the north, despite the latter two populations originating from rivers located further south (Figure 1). The differences between populations in mean latitudinal distribution decreased in the larger size classes (> 30–50 cm), but still also for these size classes, salmon from Ljusnan, Dalälven and Ljungan were caught mostly to the north. This suggests that the differences in distribution patterns among populations are governed by more factors than location of river mouth and migration speed during the first year at sea. In addition, even in the largest size class (90–110 cm), population‐specific differences in distribution were evident (Figure 1 and Table 1).

**Table 1:** Mean (± SD) recapture latitude (°N) of 10 Baltic *Salmo salar* populations caught 1951–1999, sorted north (top) to south (bottom) based on river outlet location

Population	Fork‐length size class (cm)			
	30–50	50–70	70–90	90–110
Torneälven	–	57.3 (± 2.2) ^abd^	↓ 56.4 (± 1.5) ^ab^	–
Luleälven	60.4 (± 3.1) ^a^	57.6 (± 2.5) ^a^	56.9 (± 2.0) ^ab^	56.8 (± 2.0) ^bc^
Skellefteälven	60.6 (± 2.8) ^abc^	57.5 (± 2.2) ^ab^	56.7 (± 1.8) ^ab^	56.4 (± 1.9) ^ab^
Umeälven	↓ 58.4 (± 2.8) ^d^	56.8 (± 1.8) ^b^	56.5 (± 1.6) ^a^	↓ 56.2 (± 1.4) ^a^
Ångermanälven	58.5 (± 2.5) ^d^	57.3 (± 2.0) ^ab^	56.7 (± 1.8) ^ab^	↓ 56.2 (± 1.6) ^ab^
Indalsälven	59.2 (± 2.7) ^bd^	57.4 (± 2.1) ^ab^	56.8 (± 1.9) ^ab^	↓ 56.2 (± 1.6) ^a^
Ljungan	59.7 (± 2.8) ^abd^	↑ 58.6 (± 2.3) ^c^	57.3 (± 2.1) ^bc^	↓ 56.2 (± 1.8) ^ab^
Ljusnan	61.6 (± 2.3) ^ac^	57.8 (± 2.2) ^acd^	57.2 (± 2.1) ^bc^	56.7 (± 1.7) ^abc^
Dalälven	↑ 61.9 (± 2.1) ^c^	58.3 (± 2.3) ^cd^	↑ 57.5 (± 2.1) ^c^	↑ 57.3 (± 2.1) ^c^
Mörrumsån	–	↓ 56.7 (± 1.4) ^b^	56.5 (± 1.3) ^ab^	↓ 56.2 (± 1.2) ^abc^

### 3.2 Differences in year‐class distribution variation between populations

**Figure 2:**
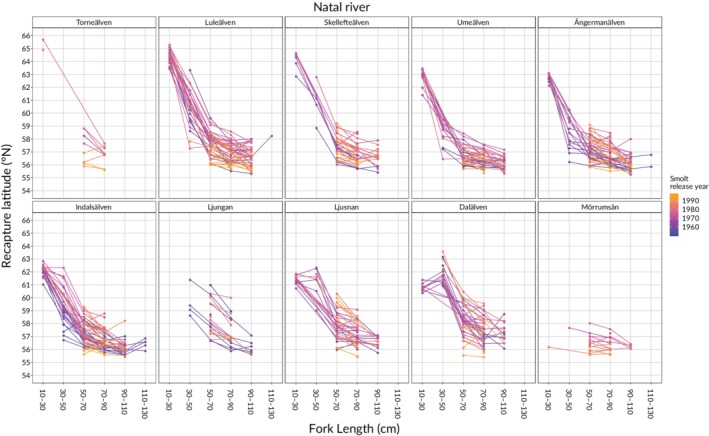
The mean recapture latitude of different smolt year‐classes for 10 Baltic *Salmo salar* populations caught 1951–1999 as a function of fork length. A year class (represented by a line) consists of tagged salmon released in the same year and river (release year indicated by colour). A large range of recapture latitudes (*i.e*., vertical range) indicates a large difference in distribution at sea among year‐classes. Smolt release year

The variation in distribution among year‐classes differed between populations in the size classes 50–70 cm and 70–90 cm (Figure 2 and Table 2). Generally, the distribution variation among year classes decreased with increasing body size (Figure 2). The largest variation among year classes was observed in the size range 30–70 cm for salmon from the rivers Luleälven, Ångermanälven and Indalsälven, but in the size range 50–90 cm for salmon from the rivers Ljusnan and Dalälven (Figure 2).

**Table 2:** Summary of the Levene's test for homogeneity of variance in distribution variation among year classes within size classes across eight Baltic *Salmo salar* populations caught in 1951–1999 (Torneälven and Mörrumsån excluded due to low sample size)

Summary statistics	Fork‐length size–class (cm)			
	30–50	50–70	70–90	90–110
*F*(*df*)	0.703 (7)	2.201 (7)	2.528 (7)	1.615 (7)
*P* *	>0.05	<0.05	<0.05	> 0.05

* Significant differences (*P* < 0.05) indicate population–specific differences in distribution variation among year–classes.

### 3.3 Individual variation in distribution

There was large individual variation in distribution among individuals from the same river also when released in the same year (Table 3, Supporting Information Figure S5). The degree of individual variation in distribution within year classes differed between populations (ANOVA, *F*_1,9_ = 15.889, *P* < 0.001) and size‐classes (ANOVA, *F*_1,3_ = 81.049, *P* < 0.001). Also, this size‐dependency differed between populations (interaction term, ANOVA, *F*_1,24_ = 3.397, *P* < 0.001). For all populations except Dalälven (Torneälven and Mörrumsån excluded due to low sample size), the largest individual variation in distribution was observed in the size class 30–50 cm, after which it generally decreased with increasing size (Table 3 and Supporting Information Figure S5.

**Table 3:** Mean standard deviation (± SD) in recapture latitude (°N) among individuals within year classes of different size classes of 10 Baltic *Salmo salar* populations caught 1951–1999. The populations with the smallest and largest degree of individual variation in distribution are denoted in bold

Population	Fork‐length size class (cm)			
	30–50	50–70	70–90	90–110
Torneälven	–	2.2 (± 1.0) ^a bc^	1.8 (± 0.9) ^a bc^	–
Luleälven	**3.1** (± 0.6) ^a^	**2.4** (± 0.6) ^a^	2.0 (± 0.5) ^a^	1.9 (± 0.8) ^a b^
Skellefteälven	3.0 (± 0.7) ^a^	2.2 (± 0.6) ^a b^	1.8 (± 0.5) ^a b^	1.9 (± 0.8) ^a b^
Umeälven	2.9 (± 0.2) ^a^	1.7 (± 0.4) ^cd^	1.5 (± 0.5) ^bc^	**1.4** (± 0.6) ^a^
Ångermanälven	2.7 (± 0.5) ^a^	2.0 (± 0.5) ^bc^	1.8 (± 0.5) ^a bc^	1.5 (± 0.6) ^a^
Indalsälven	2.7 (± 0.6) ^a^	2.1 (± 0.5) ^a b^	1.8 (± 0.5) ^a b^	1.5 (± 0.6) ^a^
Ljungan	**3.1** (± 0.6) ^a^	**2.4** (± 0.4) ^a b^	**2.2** (± 0.5) ^a^	1.7 (± 0.7) ^a b^
Ljusnan	2.5 (± 0.5) ^a b^	2.2 (± 0.6) ^a b^	2.1 (± 0.6) ^a^	1.8 (± 0.4) ^a b^
Dalälven	**2.0** (± 0.6) ^b^	2.2 (± 0.5) ^a b^	2.1 (± 0.6) ^a^	**2.2** (± 0.4) ^b^
Mörrumsån	–	**1.3** (± 0.4) ^d^	**1.3** (± 0.4) ^c^	1.7 (± 0.4) ^a b^

Different superscript letters denote significant differences in recapture latitude between populations within each size class, *P* < 0.05.

## 4 DISCUSSION

In this study, we show significant differences in size‐specific distribution patterns both between and within Swedish Baltic salmon populations. Most salmon migrate to the southern Baltic Sea for feeding, but the extent of this southward migration varies both with origin of population and body size. We also demonstrate that the populations differ in how variable their distributions at sea are, both between smolt year classes and among individuals within smolt year classes. These findings suggest that Baltic salmon populations may experience very different environments at sea, including different exploitation rates (currently assumed to be idential among populations in the Baltic salmon assessment model; ICES, 2015, 2018).

We found substantial size‐specific differences in the mean latitudinal distribution between populations. Interestingly, the most northerly originating populations reached the southern feeding grounds of the Baltic Sea first (at smallest size). In contrast, the more southerly originating populations (*i.e*., salmon from River Dalälven and Ljusnan) first perform a northward migration, followed by a migration to the southern parts of the Baltic Sea. Despite that, salmon from all 10 populations eventually reach the southern Baltic Sea, population‐specific differences in latitudinal distribution remain also among the largest individuals. Similar to our findings, previous studies on the distribution of salmon at sea originating from Finnish rivers have shown that different salmon populations can feed in different areas of the Baltic Sea (Kallio‐Nyberg *et al*., 1999, Kallio‐Nyberg & Ikonen, 1992, Torniainen et al., 2013, 2017). Our study complements these earlier studies, showing that distribution at sea varies with body size, both within and between populations. Why individuals of different body size feed in different areas of the Baltic Sea could be due to shifts in abiotic (*e.g*., temperature preference; Barbeaux & Hollowed, 2018, Morita *et al*., 2010, Otero *et al*., 2014) and biotic (*e.g*., prey availability; Jacobson *et al*., 2018, Kallio‐Nyberg *et al*., 1999) requirements over ontogeny. Size‐specific differences in distribution are important to consider as body size is a key trait governing how fish interact with prey (Jacobson *et al*., 2018, Mittelbach & Persson, 1998, Scharf *et al*., 2000), mortality risk (Lundvall *et al*., 1999, Sogard, 1997) and recruitment to size‐selective fisheries. That individuals from different populations occupy and feed in different areas at sea, even when of similar size, could be due to genetically controlled distribution patterns (Kallio‐Nyberg & Ikonen, 1992, Putman *et al*., 2014, Quinn *et al*., 2011, Royce *et al*., 1968); *e.g*., *via* evolutionary adaption to local feeding conditions (Fraser *et al*., 2011). Our analyses also give evidence for population‐specific patterns in degree of distribution variation between smolt year classes. This indicates that the distribution of some populations might be more influenced by environmental (*e.g*., currents, temperature; Ikonen, 2006, Lacroix, 2013a) and biotic drivers (*e.g*., prey availability; Mäntyniemi *et al*., 2012), compared with others (Freshwater *et al*., 2019). Also, hatchery practices (*e.g*., size at release) could differ among our study populations; these have been shown to affect the feeding distribution of Finnish Baltic salmon populations (Jutila *et al*., 2003, Kallio‐Nyberg et al., 2011, 2015, Salminen *et al*., 1994) and the time spent at sea before returning to spawn (Kallio‐Nyberg *et al*., 2011, Orell *et al*., 2018). Thus, the size at release probably affects the feeding distribution of hatchery reared Baltic salmon. The mean release size of tagged smolts varied among years and increased during our study period but was generally similar for most populations, with the largest difference between Umeälven and Dalälven (Supporting Information Figures S6 and S7). Thus, different hatcheries practices may contribute to the observed differences in distribution at sea observed among populations. Still, the fact that we found population‐specific patterns of distribution variation among populations that did not significantly differ in smolt release size (cf. Table 1 and Supporting Information Figure S7) suggests that local adaptation may play a role in the extent populations alter their distribution in response to environmental drivers. We further show that the degree of individual distribution variation differs between populations. Thus, individuals originating from the same population, entering the sea in the same year, can experience very different local environments depending on from which population they originate. This may be caused by population‐specific differences in the extent to which spatial distribution at sea is genetically determined or governed by individual responses to environmental cues (Freshwater *et al*., 2019).

Despite population‐specific differences in distribution at sea, we found that the population‐specific distribution patterns have been rather stable over time (1950s–1999), especially for some populations (*e.g*., Umeälven; Figure 2). The latter is surprising given the dramatic changes in the Baltic Sea during the study period, including a regime shift in the offshore fish species community (Casini *et al*., 2009, Möllmann *et al*., 2009), large temporal variability in available prey for salmon (Jacobson *et al*., 2018, Kallio‐Nyberg *et al*., 1999, Mäntyniemi *et al*., 2012) and increasing sea‐surface temperatures and nutrient loadings (Reusch *et al*., 2018). This five decade long stability of observed distributions suggests population‐specific distribution differences hold over time.

The observed differences in distribution at sea among Baltic salmon populations suggest that populations are likely to respond differently to changes in sea fisheries management and environmental change. The strong north–south gradient in the Baltic Sea environment and correspondingly in species composition (Bonsdorff, 2006; HELCOM, 2009) means that the environment experienced by salmon (*e.g*., temperature, salinity, size and species‐ composition of prey; Jacobson *et al*., 2018), can be very different even for a 2° latitudinal difference in distribution (Table 1 and Figure 1). Changes in the spatial distribution of commercial and recreational fishing will also affect these populations differently, depending on their spatial overlap. Populations with a more homogeneous distribution are likely to be more negatively affected if fisheries are concentrated on their feeding area than populations with a more variable distribution. Thus, not only is it important to adapt assessment and management to population‐specific distribution patterns, but management should also aim to maintain the diversity in migration and distribution patterns observed within and among salmon populations. It is increasingly recognised that such intraspecific diversity may be equally important as diversity among species to maintain ecological resilience (Schindler *et al*., 2010) and ecosystem services (Des Roches *et al*., 2018). The information on population‐specific distribution patterns provided herein is therefore important for implementation of population‐specific assessment and management of Baltic salmon also at sea. Specifically, accounting for population‐specific distribution patterns could be one way forward towards better estimates of population‐specific exploitation rates at sea (Chaput, 2012, ICES, 2018, Koljonen, 2006, Whitlock *et al*., 2018). Population‐specific responses to environmental change could also be a reason why some Baltic salmon populations have more synchronous dynamics than others (McKinell & Karlström, 1999). Thus, we argue that this distribution variation should be accounted for to better understand how Baltic salmon populations respond to changes in exploitation rates and environmental conditions at sea, as different populations clearly experience different environments at sea.

Recapture data of tagged individuals provides a snap‐shot in time of where an individual is feeding. Ideally, mark–recapture data should be combined with data on individual migratory patterns (*e.g*., using archival tags; Strøm *et al*., 2017) to further increase our understanding of the distribution patterns of different Atlantic salmon populations at sea. A potential caveat when using recaptures of tagged individuals is that in areas with salmon but no fishing, there will be no recaptures. An alternative is to use fisheries independent methods, such as stable‐isotope analyses from tissue samples of returning spawners (Dempson *et al*., 2010, MacKenzie *et al*., 2012, Torniainen *et al*., 2013). Nevertheless, according to Torniainen *et al*. (2013), assessing population‐specific distribution based on recaptures of individual salmon at sea provides similar results on a coarse spatial scale (feeding in either the northern or southern Baltic Sea) as retrospective distribution analyses using stable isotopes, collected from returning adults caught prior to spawning in their natal river. In addition, Carlin‐recaptures give an exact location of an individual (when caught), not possible to determine using retrospective distribution analyses based on stable isotopes (Hutchinson & Trueman, 2006). Even so, using recapture data when there is spatial and temporal variation in fishing effort makes it difficult to analyse changes in distribution for specific populations over time. However, we focus on comparing distributions between populations. As we only compare recaptures of equally sized individuals for the same time period at sea, we argue that the population‐specific distribution differences found in our study are caused by differences in the spatial distribution between study populations and not by spatial differences in catchabilities due to population characteristics (*e.g*., any morphological differences making individuals from some populations more likely to be caught in specific areas of the Baltic Sea compared with others). In addition, given the significant number of recaptures in this study, we argue that our estimates of differences in the distribution patterns of salmon individuals and smolt year‐classes between populations in the Baltic Sea are reliable.

In conclusion, we demonstrate large variation in size‐specific distribution patterns among and within Baltic salmon populations. These results question the assumption currently used in Baltic salmon assessment of identical responses to changes in offshore sea fisheries. The observed differences in distribution could also affect salmon population dynamics and contribute to explaining why some populations have more synchronous dynamics than others. We found consistent differences in distribution pattern between study populations over several decades, despite large‐scale changes in the Baltic Sea offshore environment. Thus, we argue that our results are important to consider in the future development of Baltic salmon assessment and management as salmon from different populations evidently experience different local environmental conditions and exploitation rates at sea. Specifically, we argue that it is key to account for distribution differences between populations at sea to succeed in current efforts to develop a more population‐specific assessment and management of Atlantic salmon in the Baltic Sea.

## CONTRIBUTIONS

All authors conceived the ideas and research questions; P.J. assembled and analysed the data; P.J. led the writing of the manuscript. All authors contributed critically to drafts and gave final approval for submitting.

## Supporting information


**FIGURE S1.** Photos showing (a) a Carlin‐tagged sea trout *Salmo trutta* smolt; (b) unique serial number; (c) instructions on the Carlin‐tag; (d) a recaptured Carlin‐tagged adult Baltic salmon *Salmo salar*.

**FIGURE S2.** Size‐specific recapture proportions of nine different Swedish Baltic *Salmo salar* populations in 2004–2010 (418 individuals) in the Baltic Sea, sorted from north (left) to south (right) based on the river mouth location. Numbers in each plot refer to the total number of recaptures for each length class and population.

**FIGURE S3.** Map showing the recapture zones in the Baltic Sea used for classifying *Salmo salar* recapture locations of reported catches in the Swedish Carlin tagging programme.

**FIGURE S4**. Size‐specific recapture proportions of 10 different Swedish Baltic *Salmo salar* populations 1951–1999 (125,432 individuals) in the Baltic Sea, sorted from north (left) to south (right) based on the river mouth location. Numbers in each plot refer to the total number of recaptures for each length class and population.

**FIGURE S5.** Mean standard deviation in recapture latitude among individuals within smolt year classes (represented by a line) for different size classes in different Baltic *Salmo salar* populations caught 1951–1999. A large vertical range of the SD indicates large year‐to‐year differences in degree of individual variation.

**FIGURE S6.** *Salmo salar* smolt year‐class specific mean length (± SD) at tagging for 10 Baltic salmon populations in 1950–1999.

**FIGURE S7.** Boxplots (–, median; interquartile range; , 95% range; |, outliers; each data point shows the mean length at tagging for each cohort) showing *Salmo salar* smolt year‐class specific mean length at tagging for 10 Baltic salmon populations 1950–1999. Different letters denote significantly different (*P* < 0.05) mean lengths at tagging between populations.

**TABLE S1.** Summary of the recaptures within smolt year classes for each length class and population used in our statistical analyzes. Total recaptures denote the total amount of recaptures for each length class and population. Maximum recaptures show the highest recaptures within one smolt year class for each length class and population. Mean and median recaptures show the mean and median number of recaptures within smolt year classes for each length class and population. Minimum recaptures show the lowest recaptures within one smolt year class for each size‐class and population. As we did not use distribution estimates based on recaptures of smolt year classes with <10 recaptures, 10 is the lowest possible number of recaptures within a specific length class for each population (for all recaptures see Figure S4).
