# Supplementary material for: Population and size‐specific distribution of Atlantic salmon Salmo salar in the Baltic Sea over five decades
Source: J Fish Biol. 2019 Dec 17;96(2):408–17. doi: 10.1111/jfb.14213 (PMC7028083; doi:10.1111/jfb.14213)
Supplement: Supplementary file 1 — FIGURE S1. Photos showing (a) a Carlin‐tagged sea trout Salmo trutta smolt; (b) unique serial number; (c) instructions on the Carlin‐tag; (d) a recaptured Carlin‐tagged adult Baltic salmon Salmo salar. [file JFB-96-408-s001.docx]

**APPENDIX S1**


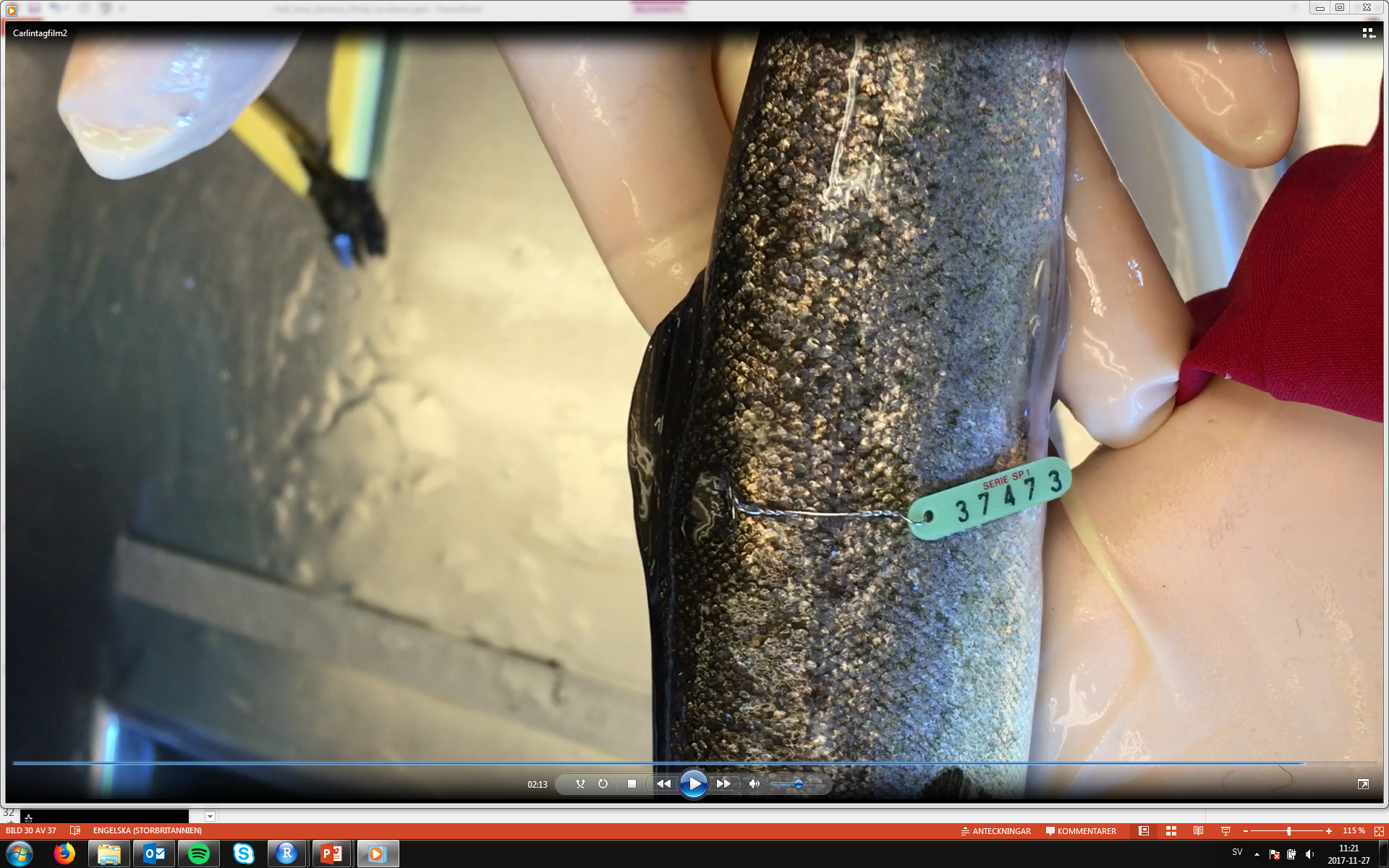

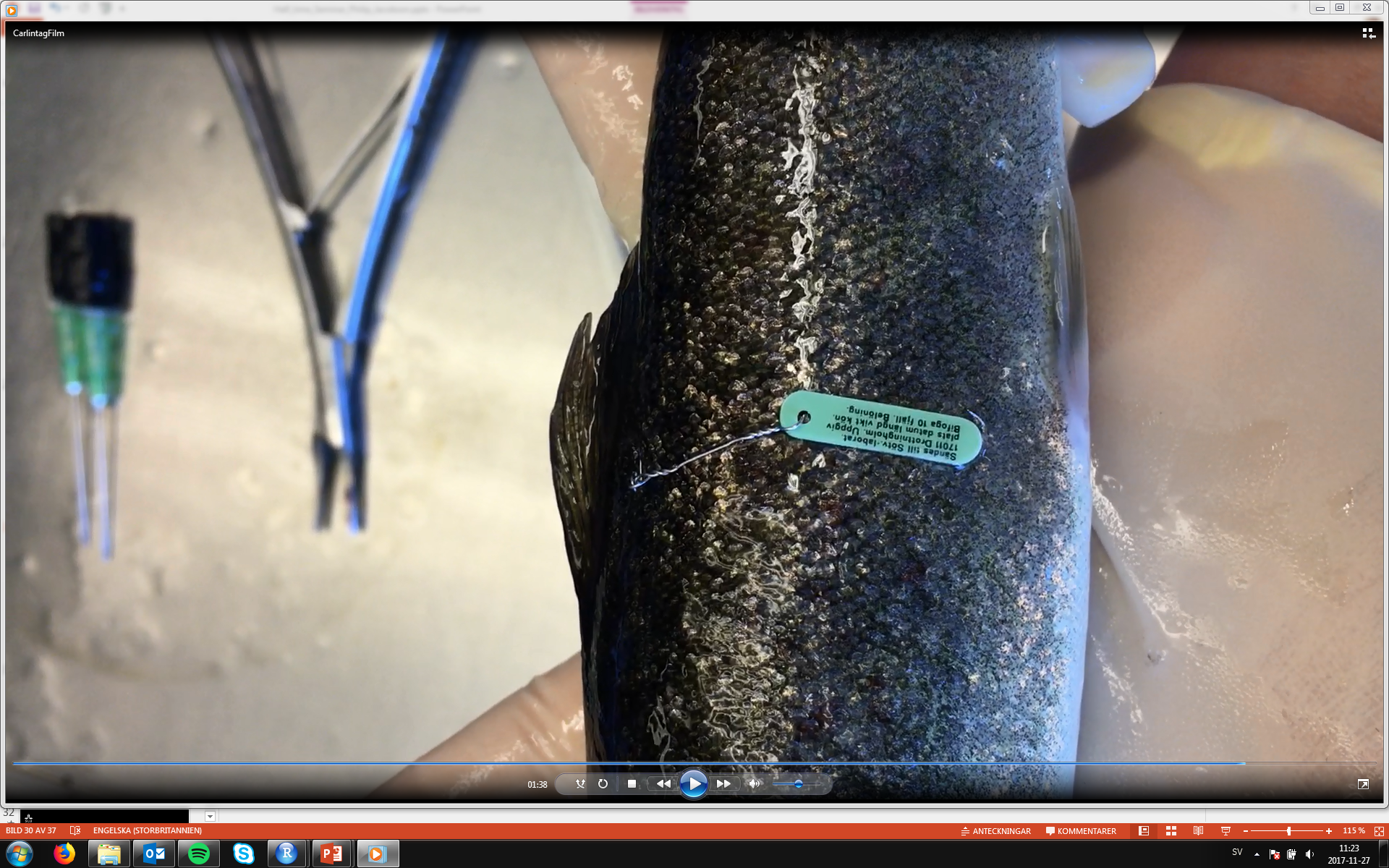

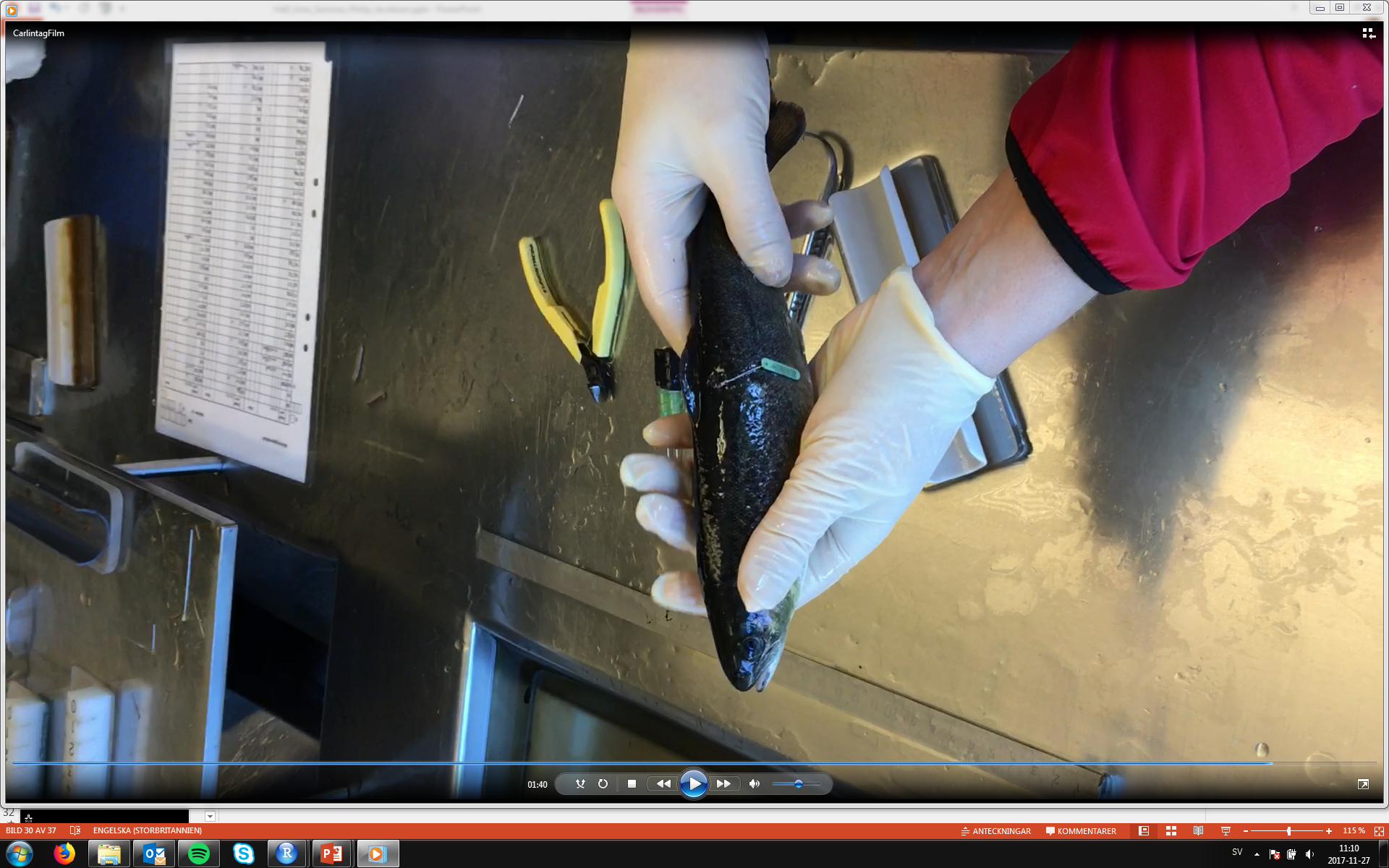


**a)**

**b)**

**c)**


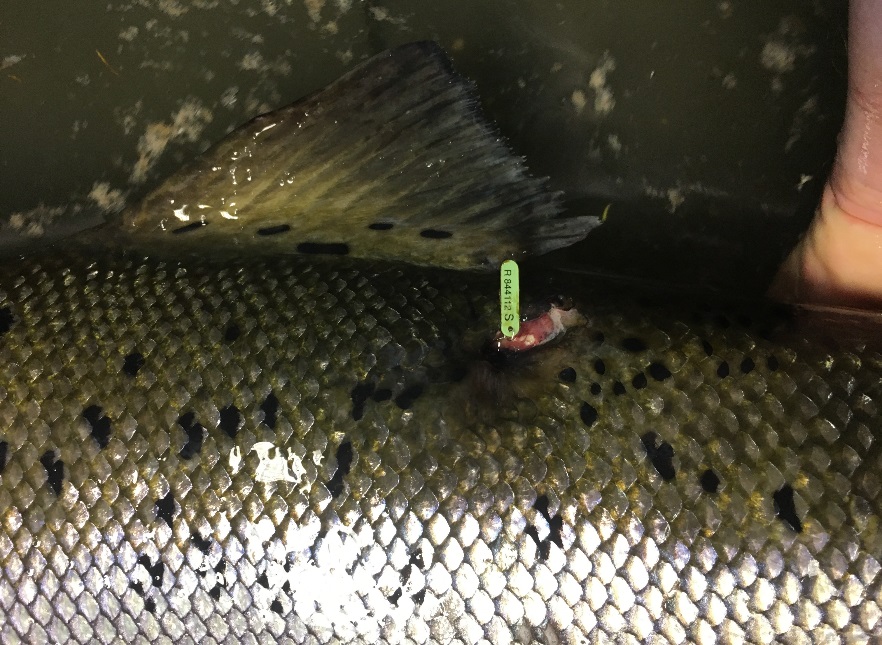


**d)**

**d)**

Figure S1. Photos showing **a)** a Carlin-tagged sea trout (*Salmo trutta*) smolt, **b)** unique serial number and **c)** instructions on the Carlin-tag and **d)** a recaptured Carlin-tagged adult Baltic salmon (*Salmo salar*).
