# Supplementary material for: Population and size‐specific distribution of Atlantic salmon Salmo salar in the Baltic Sea over five decades
Source: J Fish Biol. 2019 Dec 17;96(2):408–17. doi: 10.1111/jfb.14213 (PMC7028083; doi:10.1111/jfb.14213)
Supplement: Supplementary file 2 — FIGURE S2. Size‐specific recapture proportions of nine different Swedish Baltic Salmo salar populations in 2004–2010 (418 individuals) in the Baltic Sea, sorted from north (left) to south (right) based on the river mouth location. Numbers in each plot refer to the total number of recaptures for each length class and population. [file JFB-96-408-s002.docx]

**Appendix S3**

Recaptures of Carlin tagged salmon at sea for 2004-2010.


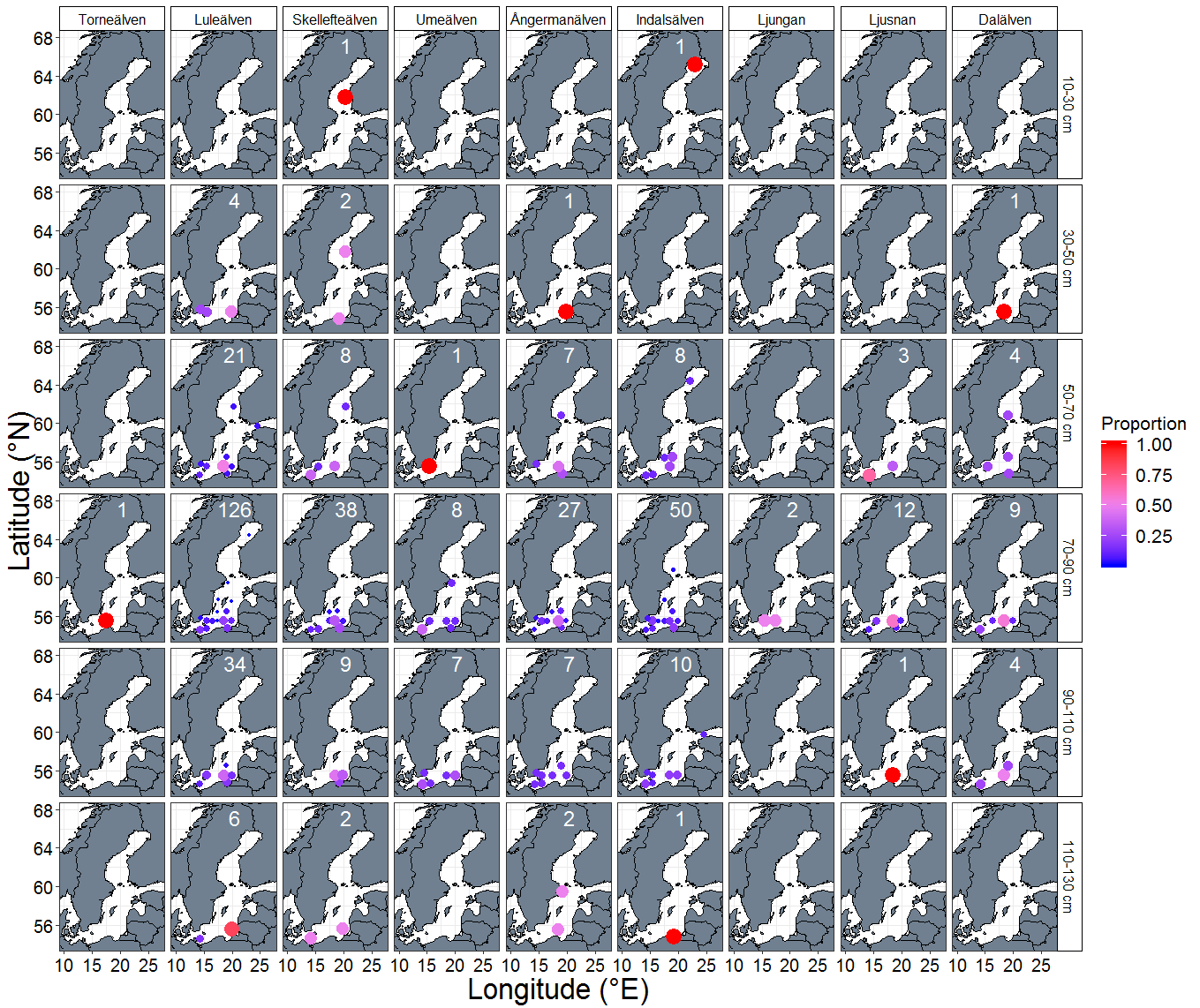


Figure S2. Size-specific recapture proportions of nine different Swedish Baltic salmon populations in 2004-2010 (418 individuals) in the Baltic Sea, sorted from north (left) to south (right) based on the river mouth location. Numbers in each plot refer to the total number of recaptures for each length class and population.
