# Supplementary material for: Population and size‐specific distribution of Atlantic salmon Salmo salar in the Baltic Sea over five decades
Source: J Fish Biol. 2019 Dec 17;96(2):408–17. doi: 10.1111/jfb.14213 (PMC7028083; doi:10.1111/jfb.14213)
Supplement: Supplementary file 3 — FIGURE S3. Map showing the recapture zones in the Baltic Sea used for classifying Salmo salar recapture locations of reported catches in the Swedish Carlin tagging programme. [file JFB-96-408-s003.docx]

**APPENDIX S2**


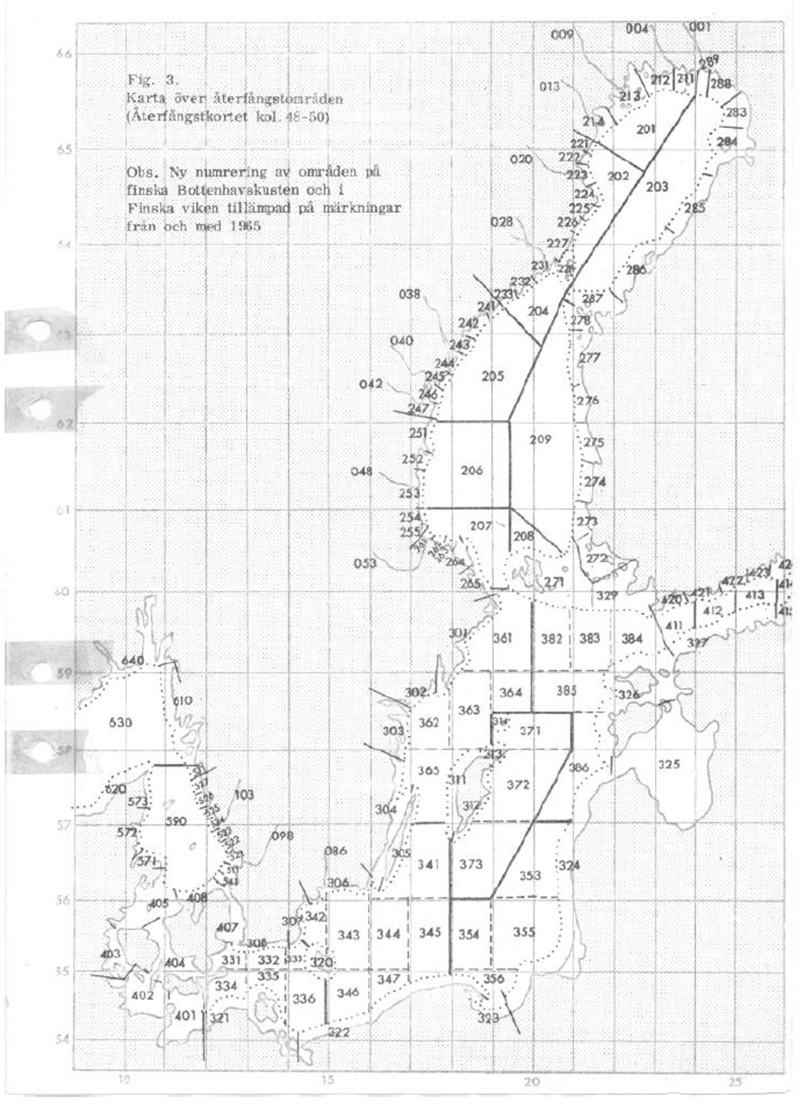


Figure S3. Map showing the recapture zones in the Baltic Sea used for classifying recapture locations of reported catches in the Swedish Carlin tagging program.


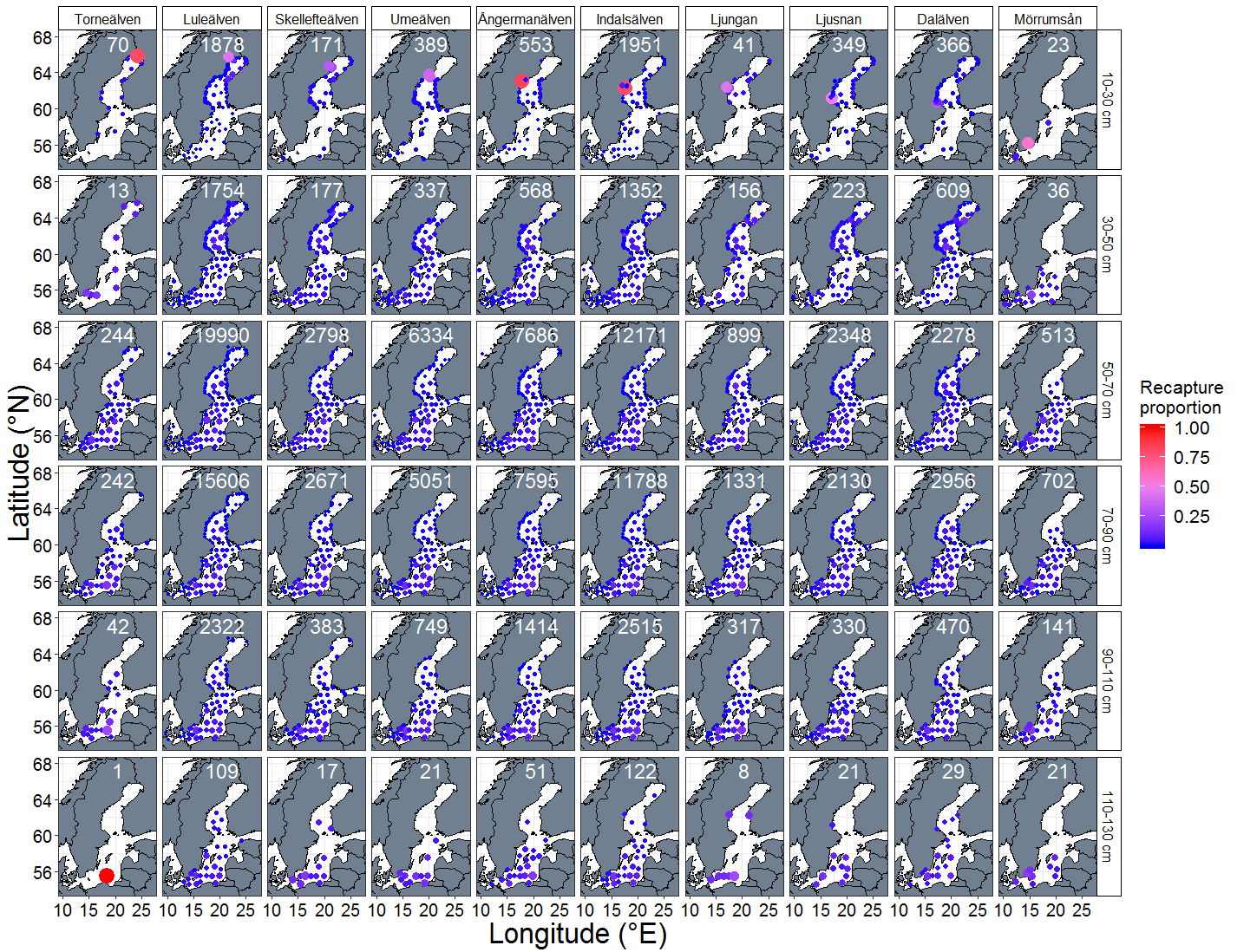


Figure S4. Size-specific recapture proportions of ten different Swedish Baltic salmon populations in 1951-1999 (125432 individuals) in the Baltic Sea, sorted from north (left) to south (right) based on the river mouth location. Numbers in each plot refer to the total number of recaptures for each length class and population.
