# Supplementary material for: Population and size‐specific distribution of Atlantic salmon Salmo salar in the Baltic Sea over five decades
Source: J Fish Biol. 2019 Dec 17;96(2):408–17. doi: 10.1111/jfb.14213 (PMC7028083; doi:10.1111/jfb.14213)
Supplement: Supplementary file 4 — FIGURE S4. Size‐specific recapture proportions of 10 different Swedish Baltic Salmo salar populations 1951–1999 (125,432 individuals) in the Baltic Sea, sorted from north (left) to south (right) based on the river mouth location. Numbers in each plot refer to the total number of recaptures for each length class and population. [file JFB-96-408-s004.docx]

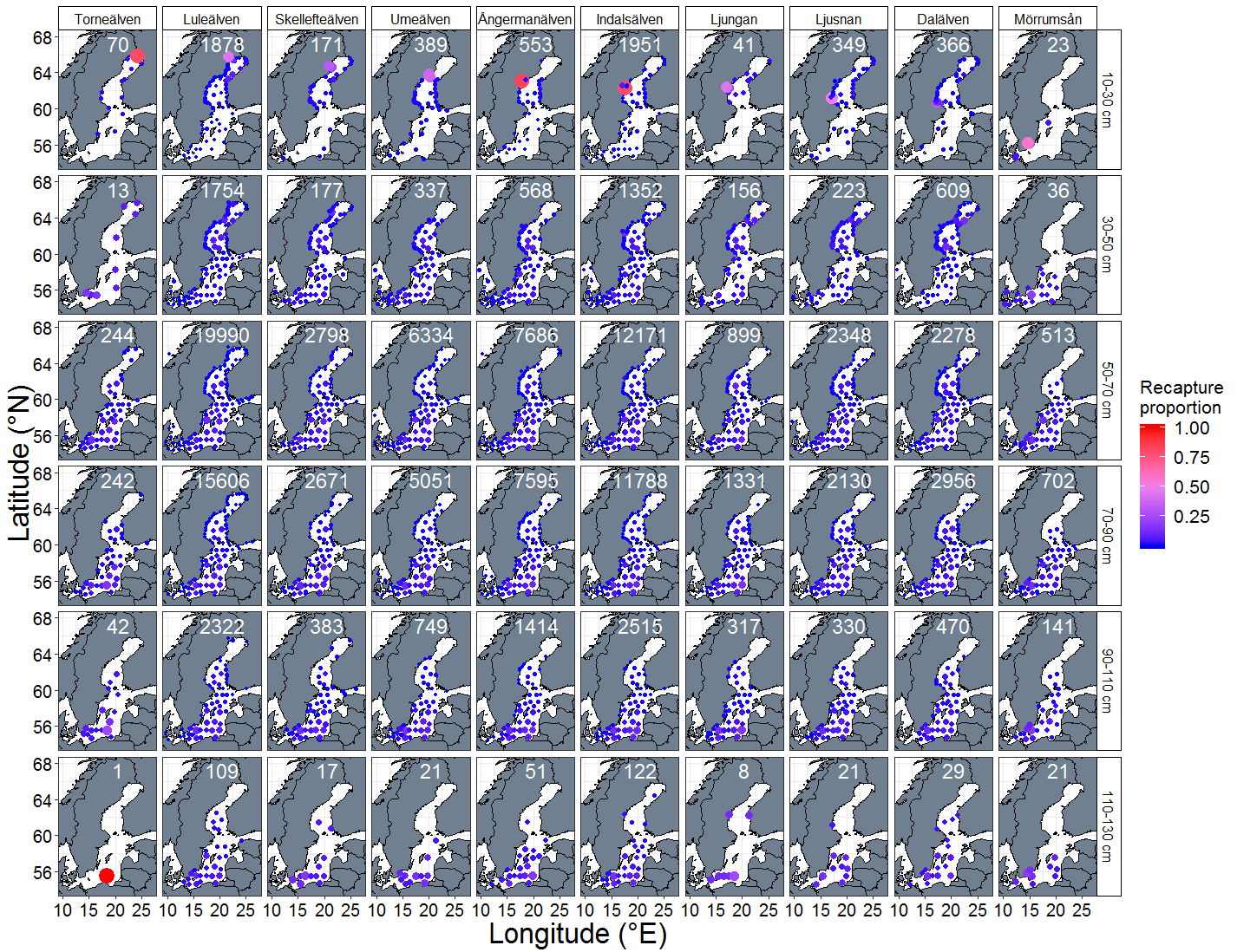


Figure S4. Size-specific recapture proportions of ten different Swedish Baltic salmon populations in 1951-1999 (125432 individuals) in the Baltic Sea, sorted from north (left) to south (right) based on the river mouth location. Numbers in each plot refer to the total number of recaptures for each length class and population.
