# Supplementary material for: Population and size‐specific distribution of Atlantic salmon Salmo salar in the Baltic Sea over five decades
Source: J Fish Biol. 2019 Dec 17;96(2):408–17. doi: 10.1111/jfb.14213 (PMC7028083; doi:10.1111/jfb.14213)
Supplement: Supplementary file 5 — FIGURE S5. Mean standard deviation in recapture latitude among individuals within smolt year classes (represented by a line) for different size classes in different Baltic Salmo salar populations caught 1951–1999. A large vertical range of the SD indicates large year‐to‐year differences in degree of individual variation. [file JFB-96-408-s005.docx]

**Appendix S4**

Figure S1


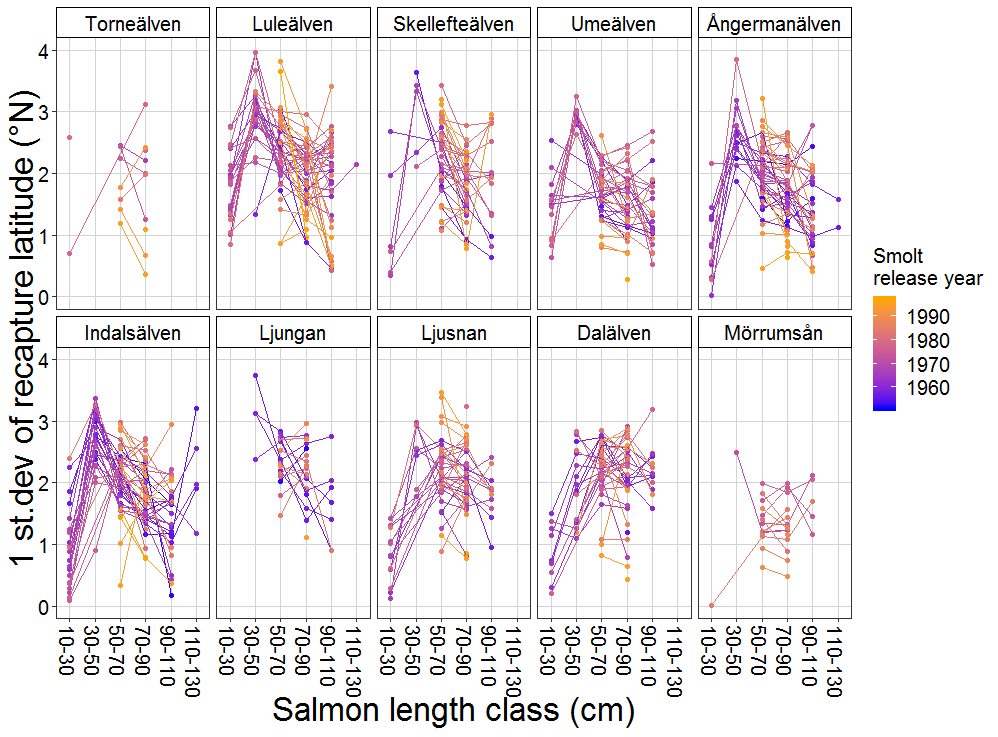


Figure S5 Mean standard deviation in recapture latitude among individuals within smolt year-classes (represented by a line) for different size-classes in ten different Baltic salmon populations caught in 1951-1999. A large vertical range of the standard deviation indicates large year-to-year differences in degree of individual variation.
