# Supplementary material for: Population and size‐specific distribution of Atlantic salmon Salmo salar in the Baltic Sea over five decades
Source: J Fish Biol. 2019 Dec 17;96(2):408–17. doi: 10.1111/jfb.14213 (PMC7028083; doi:10.1111/jfb.14213)
Supplement: Supplementary file 6 — FIGURE S6. Salmo salar smolt year‐class specific mean length (± SD) at tagging for 10 Baltic salmon populations in 1950–1999. [file JFB-96-408-s006.docx]

**Appendix S5**

Size at tagging of Carlin tagged Baltic salmon smolts in 1950-1999.


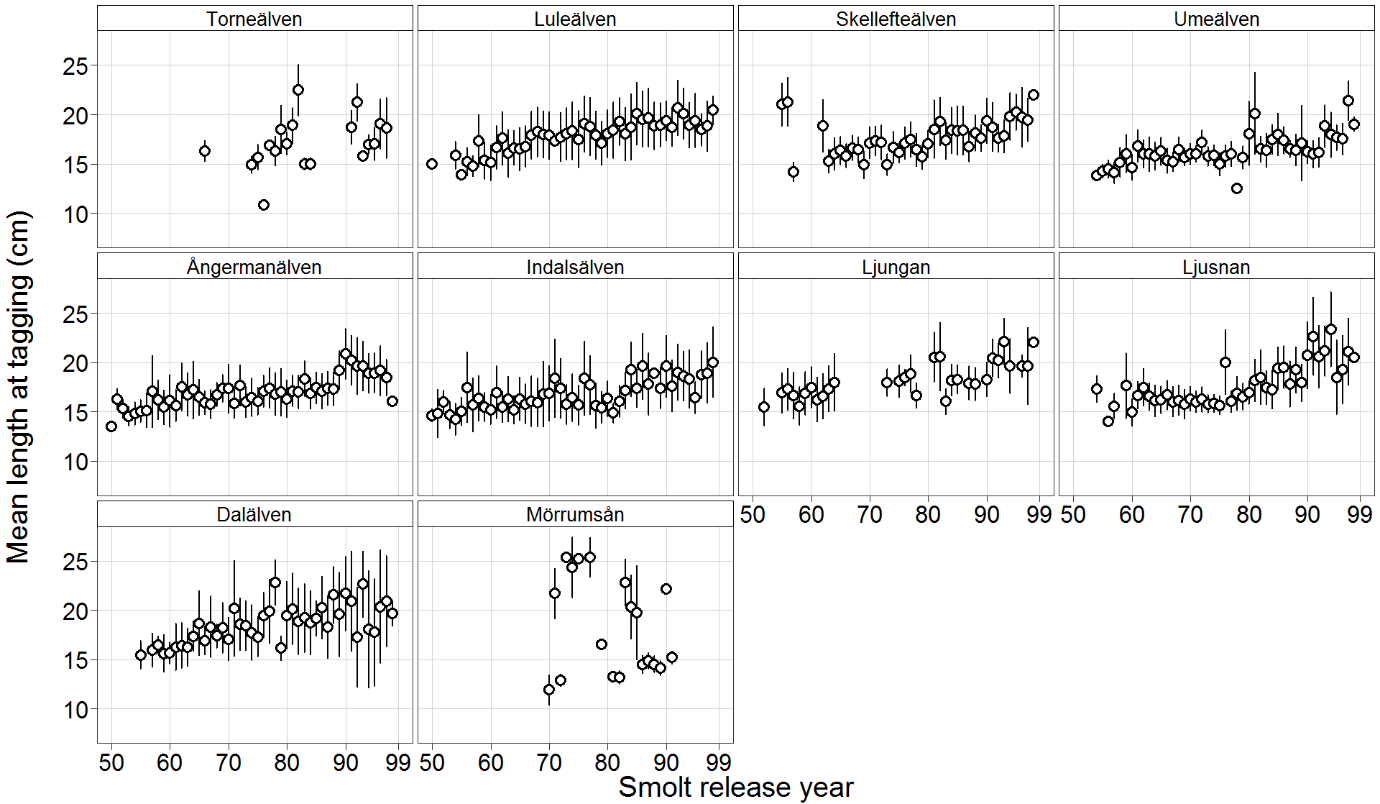


Figure S6 Smolt year-class specific mean length (±1 st.dev) at tagging for ten Baltic salmon populations in 1950-1999.


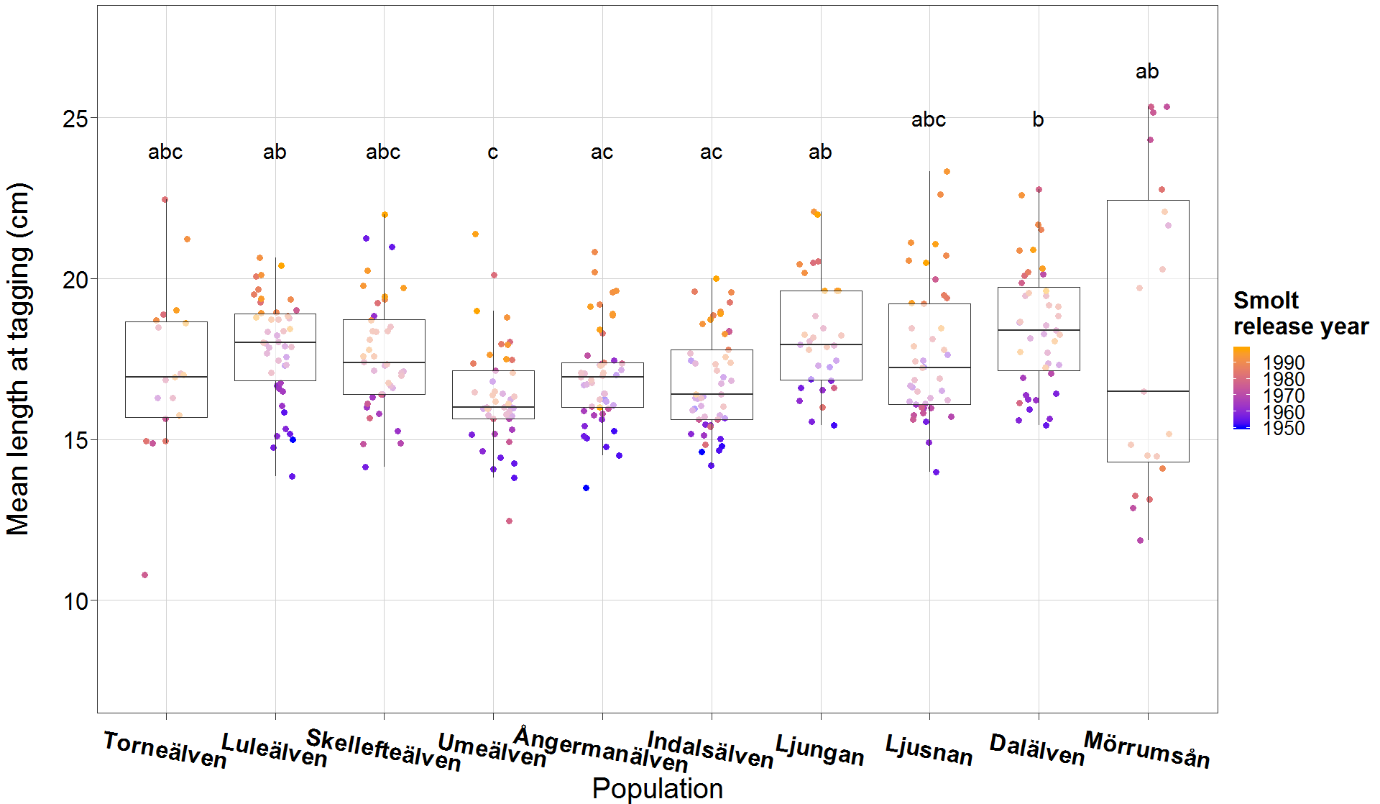


Figure S7 Smolt year-class specific mean length at tagging for ten Baltic salmon populations in 1950-1999. Each dot show the mean length at tagging for each cohort and population with corresponding boxes showing the median (solid line), the 25^th^ and 75^th^ percentile (boxes) and whiskers denote values outside the 25^th^ and 75^th^ percentile range. Different letters denote significantly different (P<0.05) mean lengths at tagging between populations, derived from one-way ANOVA followed by Tukey’s HSD post hoc test.
