# Supplementary material for: Population and size‐specific distribution of Atlantic salmon Salmo salar in the Baltic Sea over five decades
Source: J Fish Biol. 2019 Dec 17;96(2):408–17. doi: 10.1111/jfb.14213 (PMC7028083; doi:10.1111/jfb.14213)
Supplement: Supplementary file 7 — FIGURE S7. Boxplots (–, median; interquartile range; , 95% range; |, outliers; each data point shows the mean length at tagging for each cohort) showing Salmo salar smolt year‐class specific mean length at tagging for 10 Baltic salmon populations 1950–1999. Different letters denote significantly different (P < 0.05) mean lengths at tagging between populations. [file JFB-96-408-s008.docx]

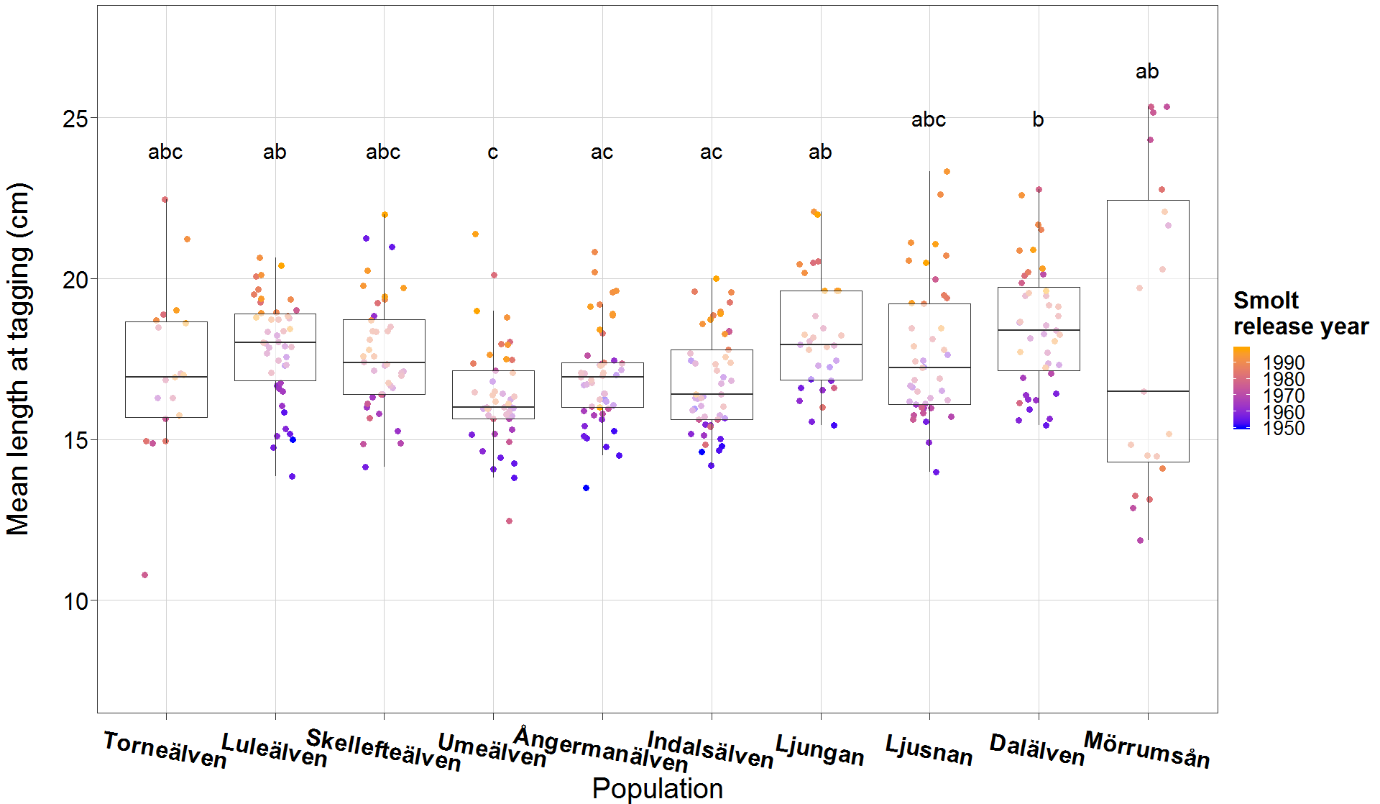


Figure S7 Smolt year-class specific mean length at tagging for ten Baltic salmon populations in 1950-1999. Each dot show the mean length at tagging for each cohort and population with corresponding boxes showing the median (solid line), the 25^th^ and 75^th^ percentile (boxes) and whiskers denote values outside the 25^th^ and 75^th^ percentile range. Different letters denote significantly different (P<0.05) mean lengths at tagging between populations, derived from one-way ANOVA followed by Tukey’s HSD post hoc test.
