# Supplementary material for: Population and size‐specific distribution of Atlantic salmon Salmo salar in the Baltic Sea over five decades
Source: J Fish Biol. 2019 Dec 17;96(2):408–17. doi: 10.1111/jfb.14213 (PMC7028083; doi:10.1111/jfb.14213)
Supplement: Supplementary file 8 — TABLE S1. Summary of the recaptures within smolt year classes for each length class and population used in our statistical analyzes. Total recaptures denote the total amount of recaptures for each length class and population. Maximum recaptures show the highest recaptures within one smolt year class for each length class and population. Mean and median recaptures show the mean and median number of recaptures within smolt year classes for each length class and population. Minimum recaptures show the lowest recaptures within one smolt year class for each size‐class and population. As we did not use distribution estimates based on recaptures of smolt year classes with <10 recaptures, 10 is the lowest possible number of recaptures within a specific length class for each population (for all recaptures see Figure S4). [file JFB-96-408-s007.docx]

**TABLE S1** Summary of the recaptures within smolt year classes for each length class and population used in our statistical analyzes. Total recaptures denote the total amount of recaptures for each length class and population. Maximum recaptures show the highest recaptures within one smolt year class for each length class and population. Mean and median recaptures show the mean and median number of recaptures within smolt year classes for each length class and population. Minimum recaptures show the lowest recaptures within one smolt year class for each size-class and population. As we did not use distribution estimates based on recaptures of smolt year classes with < 10 recaptures, 10 is the lowest possible number of recaptures within a specific length class for each population (for all recaptures see Figure S4).

| **Population** | **Length class** | **Total recaptures** | **Maximum recaptures** | **Mean recaptures** | **Median recaptures** | **Minimum recaptures** |
| --- | --- | --- | --- | --- | --- | --- |
| Torneälven | 10–30 | 29 | 16 | 15 | 15 | 13 |
| Torneälven | 50–70 | 201 | 55 | 25 | 19 | 10 |
| Torneälven | 70–90 | 206 | 39 | 21 | 18 | 12 |
| Luleälven | 10–30 | 1854 | 265 | 84 | 48 | 13 |
| Luleälven | 30–50 | 1704 | 209 | 68 | 56 | 10 |
| Luleälven | 50–70 | 19981 | 2064 | 465 | 270 | 10 |
| Luleälven | 70–90 | 15596 | 1584 | 363 | 220 | 18 |
| Luleälven | 90–110 | 2284 | 195 | 62 | 34 | 10 |
| Luleälven | 110–130 | 12 | 12 | 12 | 12 | 12 |
| Skellefteälven | 10–30 | 98 | 26 | 16 | 15 | 10 |
| Skellefteälven | 30–50 | 74 | 21 | 15 | 14 | 11 |
| Skellefteälven | 50–70 | 2782 | 242 | 75 | 66 | 10 |
| Skellefteälven | 70–90 | 2662 | 211 | 70 | 61 | 11 |
| Skellefteälven | 90–110 | 254 | 40 | 17 | 13 | 10 |
| Umeälven | 10–30 | 331 | 110 | 25 | 14 | 11 |
| Umeälven | 30–50 | 232 | 68 | 23 | 17 | 10 |
| Umeälven | 50–70 | 6321 | 1526 | 158 | 93 | 10 |
| Umeälven | 70–90 | 5038 | 789 | 126 | 76 | 10 |
| Umeälven | 90–110 | 667 | 88 | 28 | 18 | 10 |
| Ångermanälven | 10–30 | 466 | 94 | 42 | 30 | 10 |
| Ångermanälven | 30–50 | 478 | 123 | 32 | 15 | 10 |
| Ångermanälven | 50–70 | 7665 | 1655 | 170 | 94 | 11 |
| Ångermanälven | 70–90 | 7578 | 1686 | 165 | 79 | 10 |
| Ångermanälven | 90–110 | 1334 | 366 | 44 | 17 | 10 |
| Ångermanälven | 110–130 | 26 | 16 | 13 | 13 | 10 |
| Indalsälven | 10–30 | 1915 | 959 | 87 | 29 | 10 |
| Indalsälven | 30–50 | 1281 | 285 | 51 | 21 | 11 |
| Indalsälven | 50–70 | 12166 | 1592 | 259 | 132 | 11 |
| Indalsälven | 70–90 | 11784 | 1626 | 251 | 93 | 13 |
| Indalsälven | 90–110 | 2393 | 386 | 85 | 24 | 11 |
| Indalsälven | 110–130 | 67 | 16 | 13 | 15 | 10 |
| Ljungan | 30–50 | 105 | 44 | 26 | 25 | 11 |
| Ljungan | 50–70 | 862 | 125 | 43 | 25 | 10 |
| Ljungan | 70–90 | 1278 | 271 | 67 | 26 | 10 |
| Ljungan | 90–110 | 269 | 74 | 38 | 38 | 11 |
| Ljusnan | 10–30 | 292 | 60 | 21 | 17 | 10 |
| Ljusnan | 30–50 | 133 | 33 | 17 | 12 | 10 |
| Ljusnan | 50–70 | 2322 | 215 | 66 | 58 | 11 |
| Ljusnan | 70–90 | 2116 | 274 | 56 | 41 | 10 |
| Ljusnan | 90–110 | 224 | 50 | 19 | 14 | 10 |
| Dalälven | 10–30 | 310 | 76 | 34 | 19 | 12 |
| Dalälven | 30–50 | 535 | 151 | 33 | 18 | 10 |
| Dalälven | 50–70 | 2258 | 243 | 59 | 36 | 10 |
| Dalälven | 70–90 | 2945 | 348 | 74 | 44 | 10 |
| Dalälven | 90–110 | 359 | 73 | 28 | 23 | 10 |
| Mörrumsån | 10–30 | 11 | 11 | 11 | 11 | 11 |
| Mörrumsån | 30–50 | 11 | 11 | 11 | 11 | 11 |
| Mörrumsån | 50–70 | 503 | 118 | 36 | 25 | 12 |
| Mörrumsån | 70–90 | 691 | 99 | 43 | 36 | 13 |
| Mörrumsån | 90–110 | 83 | 25 | 17 | 17 | 11 |
